# The Dawn of a New Era in Kidney Transplantation: Promises and Limitations of Artificial Intelligence for Precision Diagnostics

**DOI:** 10.3389/ti.2023.12010

**Published:** 2023-12-14

**Authors:** Andrea Peloso, Maarten Naesens, Olivier Thaunat

**Affiliations:** ^1^ Division of Transplantation, Department of Surgery, Geneva University Hospitals and Faculty of Medicine, Geneva, Switzerland; ^2^ Division of Abdominal Surgery, Department of Surgery, Geneva University Hospitals and Faculty of Medicine, Geneva, Switzerland; ^3^ Department of Microbiology, Immunology and Transplantation, KU Leuven, Leuven, Belgium; ^4^ International Center of Infectiology Research (CIRI), French Institute of Health and Medical Research (INSERM) Unit 1111, Claude Bernard University Lyon I, National Center for Scientific Research (CNRS) Mixed University Unit (UMR) 5308, Ecole Normale Supérieure de Lyon, University of Lyon, Lyon, France; ^5^ Department of Transplantation, Nephrology and Clinical Immunology, Hospices Civils de Lyon, Edouard Herriot Hospital, Lyon, France; ^6^ Lyon-Est Medical Faculty, Claude Bernard University (Lyon 1), Lyon, France

**Keywords:** artificial intelligence, kidney transplant, machine learning, deep learning, transplantation

Announced as “the most revolutionary technology in decades” [1], artificial intelligence (AI) allows for the analysis and extraction of insights from huge clinical datasets. By using AI algorithms, healthcare professionals expect to gain valuable insights into patient outcomes, identify predictive factors, and develop personalized approaches for each individual. In addition, AI also holds the promise of streamlining clinical workflows, supporting real-time decision making, and enabling more efficient use of healthcare resources. As AI technology continues to develop and infiltrate new fields of our society every day, we wanted to propose a critical appraisal and try to define, among its numerous possible applications in transplant medicine, the ones that have the capability to address existing gaps and solve unmet needs.

The widespread introduction of AI in transplant nephrology has been prompted by the ever-increasing complexity and volume of information, as well as the existence of multiple nephrology registries around the world. Since the first kidney transplant, we have witnessed a shift in therapeutic goals to achieve. Initially, most efforts were focused on obtaining good short-term outcomes. This was accomplished by refining surgical techniques, researching and learning about more effective preservation solutions, and improving immunosuppression protocols. As a result, the use of kidney transplantation as a therapeutic procedure has spread rapidly (becoming, *de facto*, a victim of its own success, with growing waiting lists), and the focus has had to shift towards long-term success. In contrast with short-term outcomes, the number and diversity of variables impacting the survival of graft and patient in the long-term (including recipient’s innate and adaptive alloimmune responses, recurrence of the initial disease, nephrotoxicity of the immunosuppressive drugs, infections, cancer, etc.) [2] complexify the decision-making process, largely explaining the relative stagnation of kidney transplantation outcomes over the last few decades [3]. Here, AI could play a crucial role since this technology unveils a tremendous potential to improve immunological donor/recipient matching, kidney graft organ preservation, ischemic/reperfusion profiles, and pharmacokinetic post-transplant surveillance, which all have long term impacts (Figure 1). In addition, AI algorithms are also theoretically capable of identifying patterns and signatures indicative of rejection or generating personalized risk scores for individual patients by integrating multiple data sources including donor and recipient demographics, clinical variables, genetic testing, laboratory results, and histopathological findings.

**FIGURE 1:**
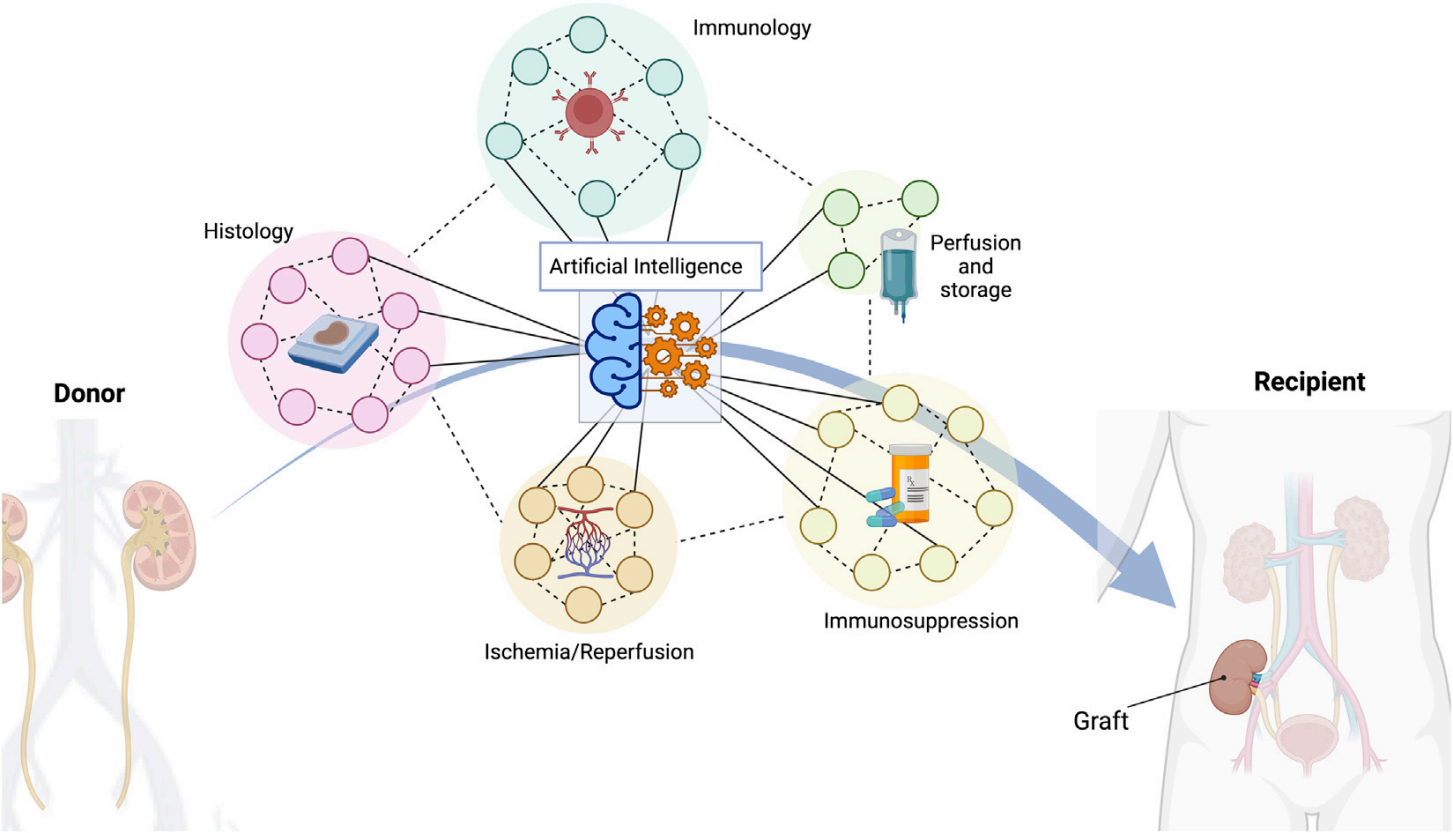
A multitude of different indicators are crucial to achieve a successful long-term outcome in kidney transplantation. Histopathological features, donor/recipient immunology matching characteristics, different types of organ perfusion and storage, several types of immunosuppressive regimes, or ischemia/reperfusion consequences are just some of the most important variables to consider. Artificial intelligence has the capacity to manage all these data and provide the best possible solution.

Biopsies provide key prognostic information for the health of allografts [4–6], which is essential for choosing the appropriate therapeutic interventions and predicting long-term outcomes. The complex and time-consuming process of histopathological analysis for kidney allograft biopsies relies heavily on the expertise of renal pathologists, which represents a significant bottleneck due to its limited availability [7]. Even when accessible, interobserver variability and the subjective nature of traditional histopathological assessment can result in diagnostic inaccuracies and misclassifications, which in turn impact clinical decision-making [7]. AI and machine learning (ML) have emerged as promising solutions to these problems, increasing the amount of information that can be collected while decreasing workload, and increasing reproducibility of the biopsy evaluation [4]. Recent studies have demonstrated the promise of AI-based solutions in the field of kidney histopathology. In 2019, Hermsen et al. [8] proposed the first convolutional neural network (cNN) applied to kidney biopsies. By using whole slide images of stained kidney transplant biopsies, the convolutional neural network (cNN) was effectively trained to perform multi-class segmentation of kidney tissue sections. It showed excellent accuracy in tissue classification, particularly in the detection of glomeruli, and demonstrated strong associations between visually scored histological elements and network-derived measurements. This research has laid a solid foundation for AI-driven quantitative investigations in renal histopathology, facilitating the integration of deep learning into everyday diagnostics. Similarly, research conducted by Ginley et al. [9] outlined the successful application of ML and image analysis algorithms to classify biopsy samples from patients with diabetic nephropathy, demonstrating substantial concordance with classifications made by three different pathologists. This study highlighted the potential of computational methods, emphasizing that these tools can provide consistent, precise interpretation of renal biopsies, thereby improving clinical diagnostic precision and providing new insights into disease progression and prognosis. In 2022, Kers and colleagues performed a retrospective, multicenter analysis on 5,844 kidney allograft slide images from 1948 patients. CNNs were trained to categorize biopsies as normal, rejection, or other diseases. A cross-validation and an external real-world cohort (counting 1,847 and 101 patients, respectively) have been used as validation. Results showed concordance for biopsies classified as normal (AUC 0.87 [CI 0.85–0.88]), as disease (AUC 0.87 [0.86–0.88]), as other diseases AUC (0.75 [0.72–0.77]), or as rejection (AUC 0.75 [0.73–0.76]). This study showed that deep learning-based classification of transplant biopsies could support pathological diagnostics of kidney allograft rejection [10]. Lastly, Yi et al focused on using AI to classify histological kidney abnormalities to use as indicators of graft loss [11]. More specifically, a deep learning algorithm was designed to improve prediction of renal allograft failure by developing a pipeline that accurately identifies and quantifies pathology related to interstitial fibrosis, tubular atrophy, and inflammation. Once the algorithm was trained on renal graft biopsies, the deriving digital features correlated significantly with existing scoring systems. Moreover, the Interstitial and Tubular Abnormality Score (ITAS) in baseline samples and the Composite Damage Score in post-transplant biopsies were highly predictive of graft loss, outperforming conventional scores or clinical predictors. Although promising, all of these examples of automated image analysis platforms are not yet ready for routine clinical implementation and several hurdles need to be overcome [12, 13].

Taking one step back, not using machine learning for image analysis but for automating the rules of the Banff Classification applied to individual lesion scores from pathology reports, Yoo et al. [14] respond to the demand for more reliable and uniform classification of kidney transplant biopsies. This system utilizes an algorithm that encodes Banff 2019 classification rules. The algorithm is embedded in an accessible, user-friendly online tool that categorizes cases into the different Banff diagnostic groups. The authors compared the system’s diagnostic accuracy, repeatability and efficiency with those of experienced pathologists from 20 transplant referral centers from Europe and North America. In the group of adult kidney transplant recipients, the Banff Automation System reassigned 83 of 279 cases of antibody-mediated rejection and 57 of 105 cases of T-cell-mediated rejection, applying the Banff rules strictly and thus more correctly than the expert pathologists did, possibly because, in day-to-day routine, pathologists draft their report before some key clinical information are available (DSA screening, etc.). A key finding of the study was the association between the system’s correction of diagnostic inaccuracies and improved assessment of long-term risks to allograft outcomes. Based on these results the authors claimed that this system has the potential to streamline study comparisons and reduce healthcare costs by preventing misdiagnoses.

In the same line [15], provides an innovative AI-based approach to merging histological and clinical data. In this work, the authors aim to overcome the heterogeneity in the interpretation of kidney allograft biopsies by applying ML-based interpretation of pathological lesions, which is improved by combining it with clinical data. This strategy strives to shed light on clinical “cloudy” situations where pathologic condition (e.g., rejection) is never really described as “absent/present,” but as a constantly changing state. 935 biopsies were read by an expert panel of pathologist and transplant physicians. The resulting ML diagnostic classifier was then put to the test on three distinct biopsy cohorts for a total of 4,693 biopsies. The ML classifier showed remarkable consistency, achieving over 90% accuracy in predicting and diagnosing T cell-mediated rejection, antibody-mediated rejection, and interstitial fibrosis-tubular atrophy. It also showed superior performance when compared to a computer-based decision algorithm that strictly adhered to the Banff rules without taking clinical context into account. Therefore, the use of AI, integrated with clinicopathological features, can significantly improve diagnostic efficiency. Notably, the classifier showed perfect accuracy in categorizing six cases previously highlighted in a Banff Working Group survey [13] involving 72 pathologists and 95 clinicians, which demonstrated that the human participants deviated from the reference diagnosis in 26% and 35% of cases for pathologists and clinicians, respectively.

Next to the evaluation of biopsies and rejection diagnosis, other applications of AI in kidney transplantation are emerging. After kidney transplantation, recipients are monitored intensively. Every transplant center faces the challenges of a rigorous follow-up with early detection of post-transplant complications and effective management of immunosuppression. In this context, AI is increasingly becoming implemented, particularly in overseeing immunosuppression regimes via pharmacokinetics analysis and in predicting recipient pharmacokinetic behavior [16–18]. The multi-faceted nature and the inter-individual variability of immunosuppression management poses significant challenges due to the need for individualized treatment plans and vigilant monitoring to prevent rejection episodes or adverse therapy effects. Also, ML algorithms trained on historical patient data can predict individual responses to different immunosuppressive drugs, helping clinicians determine the optimal dosages and combinations of these drugs. In addition, AI tools can help monitor patient adherence to medication regimens and alert clinicians to any deviations. This can be invaluable in an area where non-adherence can have devastating consequences. For example [17], developed dose prediction algorithms to forecast recipient tacrolimus dose after kidney transplantation. This study enrolled 1,045 kidney transplant patients. Different ML models [including multiple linear regression (MLR), artificial neural network (ANN), and regression tree (RT)] were applied and evaluated. Among all ML-models, the RT model showed outperformance in both cohorts [derivation cohort 0.71 (0.67–0.76); validation cohort 0.73 (0.63–0.82)]. Moreover, RT exceeded the MLR model by 4%. This frontline paper was the first to propose using ML to predict tacrolimus stable dose. More recently [19], developed ML prediction models (Xgboost) to estimate tacrolimus inter-dose AUC based on a limited number of blood concentrations and predictors. Two different cohorts of patients have been analyzed following twice-a-day and once-a-day dosing. Every model was subjected to data division, allocating 75% for the training set and 25% for the test set. Xgboost models in the training set that exhibited the lowest Root Mean Square Error (RMSE) in a ten-fold cross-validation experiment were then assessed in the test set as well as six independent full pharmacokinetic datasets from kidney, liver, and heart transplant recipients. Xgboost models demonstrated excellent AUC estimation capabilities in the test datasets, with relative bias under 5% and relative root mean square error (RMSE) below 10%. Furthermore, these models outperformed the Maximum A Posteriori (MAP) Bayesian estimation in the six independent full pharmacokinetic datasets.

Despite the promises of AI-driven follow-up after kidney transplantation, a number of hurdles must be overcome before these systems can be implemented in clinical practice. First, to minimize biases and improve generalizability, the performance of AI algorithms is heavily dependent on the quality and quantity of data used for training [15]. Second, the integration of AI systems into clinical workflows requires validation in real-world settings and consideration of ethical, legal, and regulatory aspects [20, 21]. Additionally, it is important to remember that AI-driven histological classification systems should not be viewed as a replacement for expert renal pathologists but rather as a complementary tool that can enhance their diagnostic abilities. The combination of human expertise and AI-driven approaches can lead to improved diagnostic accuracy and better-informed clinical decision-making, which will ultimately benefit patients and the broader transplantation community [22, 23].

The application of AI is still in its early stages and, aside from ethical or privacy issues, the current hype for this technology should not overshadow AI’s intrinsic limitations. The scientific method is based on a six-step cycle: observe, define a question, predict, collect data, analyze data, and draw conclusions [24]. AI can only improve the prediction phase but lacks the potential to generate data or new hypotheses autonomously. In addition, in the prediction phase, AI should not simply be identified as an “oracle technology” that will correctly predict the outcome of an experiment. AI could create new correlations, but not causal links [25]. A full understanding of how the predictions of the “oracle” are arrived at is an integral part of scientific understanding, and therefore AI should be integrated with causal inference reasoning to be fully exploited in the future. Moreover, existing AI systems that base their predictions only on associations in data are highly vulnerable to any changes in the way these variables are related [25]. That is why, at this stage, AI cannot replace the nuanced and complex decision-making skills inherent in transplant medicine.

## Data Availability

The original contributions presented in the study are included in the article/supplementary material, further inquiries can be directed to the corresponding author.
